# Small Peptides Compound Isolated from* Agkistrodon* with Antiarthritic Effect in Collagen-Induced Arthritis Rats

**DOI:** 10.1155/2018/8265150

**Published:** 2018-04-29

**Authors:** Lijun Mei, Chen Lin, Shanshan Lei, Li Xu, Yong-Sheng Fan

**Affiliations:** ^1^Zhejiang Chinese Medical University, Hangzhou, Zhejiang 310053, China; ^2^Zhejiang University of Technology, Hangzhou, Zhejiang 310014, China

## Abstract

*Agkistrodon* in Chinese medicine has long been used as an effective treatment against rheumatoid arthritis (RA). The present research further investigated the effects of peptides extracted from the crude* Agkistrodon* on the RA rat model. Extracted peptides were separated by parameter-optimized ion-exchange chromatography (IEC), peptide fractions were further analysed by MALDI-TOF/TOF MS, and nano-LC-MS/MS acquired mass spectra were further characterized using Mascot software, which ranks the best matches in the NCBI database. RT-PCR results in RAW264.7 cells indicated that* Agkistrodon* peptide components had inhibitory effects against inflammatory cytokines. The therapeutic efficacy of* Agkistrodon* peptides was evaluated on the Wistar rats with collagen-induced arthritis. Symptom relief and reduced cartilage destruction and bone erosion were observed, which can be explained by the direct suppression of inflammatory cytokines in the joints.* Agkistrodon* peptides downregulate the expression of TNF-*α*, IL-1*β*, and IL-6, which may alleviate cartilage destruction and bone erosion, thus relieving symptoms of RA.

## 1. Introduction

Rheumatoid arthritis (RA) is a chronic inflammatory disorder characterized by progressive destruction of patients' joints caused by cellular infiltration and proliferation of synovium [1]. Proinflammatory cytokines including TNF-*α*, IL-1*β*, and IL-6 are overproduced in the rheumatoid joints and are critical mediators in the pathogenesis of RA, promoting leukocytes, and osteoclasts activation, driving destruction of bone and cartilage [2]. Inhibiting of proinflammatory cytokines has been shown to improve disease symptomatology and outcome in rodent arthritis models and human clinical trials [3, 4].

The prognosis of RA so far is discouraging, given that common treatments including disease-modifying antirheumatic drugs (DMARDs) and nonsteroid anti-inflammatory drugs (NSAIDs) partially relieve patients' symptoms with limited and short-term effectiveness [5, 6], and prolonged usage might cause serious adverse effects such as leukopenia, elevated transaminases, and infection [7–9]. On the other hand, it is increasingly acknowledged that natural products with antiarthritic effects might be a good choice for potent and safe treatment of RA [10]. Insects as natural product resources have since ancient times been used for antiarthritic therapy [11]. Successful isolation of bioactive molecule from secretions, excretions, and bodies of insects further encourages the in-depth exploration of their functional mechanisms [12].

A wide range of small peptide toxins have been separated from venoms and larvae. Bioactive peptides inhibit synovial angiogenesis by binding to the vasculature endothelial cells [13] or suppressing immune response by inhibiting induction of T-cell mediated inflammation [14]. Recent studies searching from snake venoms have successfully identified anti-inflammatory and antiarthritic activities components and further demonstrated that their beneficial effects might be attributed to the inhibition of NF-*κ*B pathway and suppression of T-cell/FLSs proliferation [15–17]. Preclinical models of peptide immunotherapy have demonstrated efficacy in autoimmunity, shedding light on the potential as pharmaceutical agents [18, 19].

In the present study, peptides isolated from the crude medicine of snake* Agkistrodon acutus* were analysed for their potency/efficacy on/against arthritis. The dried powder of* Agkistrodon acutus* has been successfully applied in Chinese medicine as an antiarthritic drug, and previous work explored and determined the optimal treatment dosage [20]. Peptides' cytotoxicity and cytokine-inhibiting effects were tested and affirmed by both in vitro and in vivo experiments. Possible mechanism in association with the cytokine-inhibiting effect was discussed.

## 2. Materials and Methods

### 2.1. Animals and Cells

Female Wistar rats (*n* = 30, 6-7 weeks of age) were obtained from the Laboratory Animal Research Center of Zhejiang Chinese Medical University (China; rodent license number SYXK (Zhe) 2003-0184). After acclimatization for 7 days, four rats were housed in each Individual Ventilation Cage (IVC) and were maintained in accordance with national standards (Laboratory Animal Requirements of Environment and Housing Facilities, GB 14925-2001). Six rats were randomized into a normal control group and all others were used for the CIA model. RAW264.7 cells were purchased from the Shanghai Institutes for Biological Sciences, Chinese Academy of Sciences.

### 2.2. Reagents

The* Agkistrodon* for the* Agkistrodon* powder and the* Agkistrodon* peptide preparation was purchased from the Chinese Herbal Medicine Co., Ltd., of Zhejiang Chinese Medical University (Hangzhou, China). Glycoside of* Tripterygium wilfordii* (number 1507101B) was purchased from Zhejiang Deende Pharmaceutical Co., Ltd., China. Foetal bovine serum (FBS, number 10099141) was purchased from Gibco Life Technologies., Inc., USA, and high-glucose Dulbecco's modified Eagle's medium (DMEM) was purchased from Hangzhou Sijiqing Biological Engineering Materials Co., Ltd., China. The Cell Titer 96 AQueous One Solution Cell Proliferation Assay (MTS, number G3581) and lipopolysaccharide (LPS, number L2880) were purchased from Sigma, USA. TRIzol reagent (number 9109) and Prime Script RT Master Mix (number RR036A) were purchased from TaKaRa, Japan. SYBR-Green Supermix (number 170-8882AP) was purchased from Bio-Rad, USA. The specific primers for the target genes and GAPDH were synthesized by and purchased from Shanghai Shenggong Co., China. Incomplete Freund's adjuvant (IFA, number SLBK3881V) was purchased from Sigma, USA. Bovine type II collagen (CII, number 20021) and the enzyme-linked immunoassay (ELISA) kit (number 2042) were purchased from Chondrex, USA. The TNF-*α* polyclonal antibody (number YT4689) and IL-*β* polyclonal antibody (number YT5201) were purchased from ImmunoWay, USA. The IL-6 polyclonal antibody (number BS6419) was purchased from Bioworld, USA.

### 2.3. Peptide Separation

Extraction of crude peptides was processed following instructions of the boiling method with dilute ethanol recorded in the Chinese Pharmacopeia (2010 edition). Briefly, 10 grams of crude* Agkistrodon* medicine was powdered and sifted through a 149 *μ*m mesh filter and then was further dried for 30 min to remove any additional water. The powder was then soaked in 50% dilute ethanol, with its weight/volume percentage concentration to be 10%. The total crude peptides were heat reflux extracted for 1 h and then after cooling to room temperature, the solution was centrifuged at 10000 ×g for 20 min. Supernatants were collected and tested for protein/peptide concentration and then lyophilized at −56°C and stored for further tests.

Lyophilized crude peptides, 1.8 grams, were measured and dissolved in pure water. Ion-exchange chromatography was used for separation. An XK 26/20 column was packed with Resins purchased from GE, USA, and separation was performed with a 0–0.5 M NaCl gradient and monitored at a 215 nm wavelength. Eluted peptide peaks were collected and desalted by dialysis with a 500 Da cutoff before being lyophilized and further analysed by an AB SCIEX 5800 MALDI-TOF/TOF mass spectrometer (AB Sciex, Canada) in linear positive mode. Mass spectra were analysed using Data Explorer 4.5 software (Perspective Bio Systems). Spectra were acquired over the mass range of 600–4,000 Da using 50 laser shots in the positive ion mode at a laser power of 25%.

### 2.4. MALDI-TOF Analysis

The molecular mass of lyophilized peptides was analysed by MALDI-TOF/TOF on an AB SCIEX 5800 Proteomics Analyser (AB Sciex, Canada). Samples were mixed thoroughly at a ratio of 1 : 1 (v/v) with a saturated matrix solution (*α*-cyano-4-hydroxycinnamic acid, 5 mg/mL prepared in 50% acetonitrile/0.1% trifluoroacetic acid), and 0.5 *μ*l of the mixture was spotted onto a target plate. The mixture was fully dried before analysis. The instrument was calibrated externally using a mixture of peptide standards obtained from Sigma-Aldrich (MS-Cal1). Positive ion (MH+) spectra were acquired in the linear MALDI-TOF mode. The* m/z* signals were recorded between 1,000 and 10,000 Da for peptide detection.

### 2.5. LC-MS/MS Analysis

To explore and identify species-specific peptides, LC/MS/MS analysis was performed. The collected peptide sample was analysed by a Thermo Q Exactive mass spectrometer (Thermo, San Jose, CA) with a nanospray ion source. The lyophilized peptides were dissolved in FA–H_2_O (0.1 : 99.9, v/v) and preconcentrated on a 2 cm trap column (100 *μ*m i.d.) that was packed with C18 AQ beads (5 *μ*m, 120 Å, Daison, Osaka, Japan). A second 10 cm capillary analysis column (75 *μ*m i.d.) was packed with C18 AQ beads (3 *μ*m, 120 Å, Daison, Osaka, Japan) and was used to separate the peptides. Eluting buffers were 99.9% H_2_O with 0.1% FA (buffer A) and 84% ACN with 0.1% FA (buffer B). The separation system was equilibrated initially with buffer A, and a linear elution gradient (at flow rate of 320 nil/min) was performed, from 4 to 50% buffer B (formic acid-acetonitrile, 0.1 : 99.9) in 20 min and from 50% to 100% buffer B (FA-acetonitrile, 0.1 : 99.9) in 5 min, and then held at 100% buffer B for another 5 min. The MS/MS spectra were obtained in collision-induced dissociation (CID) mode, and a full mass scan was acquired from* m/z* 400 to 2000 with a resolution of 70,000. The 10 most intense ions with a charge above 2 and intensity threshold higher than 104 were selected for MS/MS. Dynamic exclusion was set at 30 s.

### 2.6. Data Analysis

Raw mass spectrometry data were compared with that of the Squamata in the NCBI database using the Mascot search algorithm (version 2.2), which ranks the best fit peptide matches based on a cumulative protein score of the unique peptides identified.

The criteria used for the Mascot search are as follows: trypsin was used as the protease with a maximum of 1 missed cleavage allowed; the protein oxidation of methionine was fixed as a dynamic modification whereas the carbamidomethyl of cysteine was retained as a static modification; and the protein list was further filtered by applying a false discovery rate cutoff of 1% at the protein level. The data were filtered for ion scores of at least 60. For each file, an ion score > 20 indicated identity or extensive homology with *P* < 0.05.

### 2.7. Cell Culture and Cytotoxicity Assay

The RAW264.7 cells were cultured in high-glucose DMEM with 10% FBS in a 37°C incubator with 5% CO_2_. The viability of RAW264.7 cells was evaluated with the MTS assay. The cells were seeded in 96-well plates at a density of 10 × 10^4^ cells/well for 24 h and then treated with various concentrations of separated peptides (0.1, 1, 10, 100, or 1000 *μ*g/mL) for 24 h. Optical density (OD) was measured at 450 nm using a Multiskan Spectrum Microplate Spectrophotometer (Bio-Rad, California, USA). Cell viability was expressed as a percentage of the control.

### 2.8. RNA Extraction and Real-Time Polymerase Chain Reaction (RT-PCR) Analysis

The RAW264.7 cells were cultured in 6-well plates at a density of 10 × 10^4^ cells/mL for 24 h. Following pretreatment with LPS (10 *μ*g/mL) for 4 h, the cells were treated with different concentrations of* Agkistrodon* peptides (1, 10, or 100 *μ*g/mL) for 24 h.

Total RNA was extracted using TRIzol reagent. Real-time PCR was performed using SYBR-Green Supermix on a Bio-Rad iQ5 Real-time PCR system (Bio-Rad, California, USA). The mouse primer sequences were used as described in Table 1. Data were calculated using the formula 2^−ΔΔCt^ and all values were normalized to the level of GAPDH.

### 2.9. Induction of Collagen-Induced Arthritis (CIA)

CII was dissolved in 100 mM acetic acid at the concentration of 4 mg/mL by stirring overnight at 4°C. Dissolved CII was emulsified with an equal volume of incomplete Freund's adjuvant (IFA) on ice using a T-branch pipe and two syringes to make the final concentration of 2 mg/mL CII/IFA emulsion. For the primary immunization, CII/IFA emulsion was injected subcutaneously at the base of the rat tail, as the first site, and on the back, as the second site, totalling 200 *μ*l of emulsion containing 400 *μ*g of CII. A booster injection was given on day 7 after the initial immunization with 100 *μ*l of the emulsion containing 200 *μ*g of CII. Visually apparent arthritis with swollen joints appeared approximately 11–13 days after the first injection.

### 2.10. Animal Grouping and Treatment

**Figure 1:**
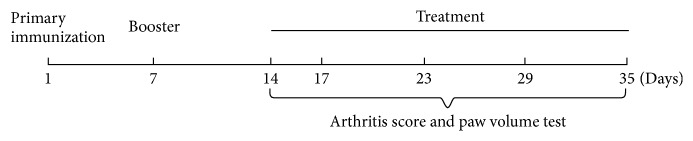
Scheme of animal experiment protocol. From day 14, rats with CIA were treated with Glycoside of* Tripterygium wilfordii*,* Agkistrodon* powder, and* Agkistrodon* peptides.

After the joints swelling was successfully established in at least one paw, the rats were randomized into groups, with the mean of their hind paw volumes and arthritis scores in each group approximately equal. Rats with CIA were randomly divided into 4 groups: Glycoside of* Tripterygium wilfordii* (GTW, 0.72 mg/kg body weight, administered by gastric gavage daily),* Agkistrodon* powder (APO, 580 mg/kg body weight, administered by gastric gavage daily),* Agkistrodon* peptides (APE, 4 mg/kg body weight, administered by intraperitoneal injection daily), and the CIA model group (Figure 1). In addition, the normal control group received nothing. In this study, there were six mice per group and a total of 5 groups.

### 2.11. Evaluation of Paw Swelling Degree

The paw swelling degree was determined by measuring the change in the hind paw volume (mL) with a plethysmometer (type: YLS-7C, Yan-yi Technology Development Co., Ltd., Jinan, China). The paw swelling rate (%) was expressed as increased multiples of the hind paw volume by subtracting the paw volume before the primary immunization.

### 2.12. Estimation of Arthritis Indexes

The development of arthritis in the hind paws of rats was evaluated before treatment and on days 14, 17, 23, 29, and 35 by using a qualitative scoring system: 0, normal; 1, mild, but definite redness and swelling of the ankle or wrist or the apparent redness and swelling are limited to individual digits; 2, moderate redness and swelling of the ankle; 3, severe redness and swelling of the entire paw including the digits; and 4, maximally inflamed limb involving multiple joints.

### 2.13. Determination of Type II Collagen IgG Antibodies

IgG antibodies against type II collagen in serum was assessed with ELISA kits. According to the manufacturer's protocol, serum samples from arthritic rats were centrifuged at 10,000 rpm for 3 minutes and diluted with sample dilution buffer from 1 : 20,000 to 1 : 80,000. The OD values were read at 490 nm using a Multiskan Spectrum Microplate Spectrophotometer (Bio-Rad, California, USA) and expressed as units per millilitre.

### 2.14. Radiographic Examination

At the end of the study (day 35), the hind limbs were collected for radiographic evaluation. X-ray radiography of the ankle joint was performed using an in vivo Imaging System FX (Carestream Health, Rochester, USA) with a 35 kW exposure for 2 min. The three-dimensional reconstruction and images were obtained by using high-resolution in vivo X-ray microtomography (Bruker, Germany).

### 2.15. Histologic Assessments

At the end of the study (day 35), the hind limbs were preserved in 10% neutral buffered formalin fixative and then placed in decalcifying solution (a mixture comprising formic acid, hydrochloric acid, and distilled water) for 50 days (decalcifying solution was exchanged every 5 days). After that, all ankles were longitudinally trimmed, embedded in paraffin, sectioned at 5 *μ*m, and stained with haematoxylin and eosin (H&E), safranin O, and toluidine blue.

### 2.16. Immunohistochemical Analysis

For immunohistochemistry, sections were prepared from the same preparations used for H&E staining. The sections were incubated with specific antibodies for rat TNF-*α*, IL-1*β*, and IL-6. Images were observed with light microscopy (Nikon, Tokyo, Japan) and representative images were revealed.

### 2.17. Statistical Analysis

Data were expressed as the mean ± SEM or mean ± SD. For comparisons among groups, a one-way analysis of variance (ANOVA) followed by a Games-Howell test was performed to test significant differences. All data were analysed using SPSS 17.0. *P* < 0.05 was considered to be statistically significant.

## 3. Results

### 3.1. *Agkistrodon* Peptides Compound Isolation and Identification

Following instructions of the Chinese Pharmacopeia (2010 edition), crude peptides were extracted and were further separated by taking advantage of ion-exchange chromatography. Initial separation using a 0–100% linear gradient of NaCl was unsuccessful (data not shown), which might be attributed to the existence of components not responsive to the NaCl gradient. The separation protocol was therefore modified by increasing the NaCl to 0.5 M after the elution of detected peaks of NaCl-unresponsive. An elution peak responding to the increased NaCl was observed, and repeat tests performed demonstrated similar results (Figure 3(a)).

The NaCl responsive eluate was collected and further dialyzed for mass determination by MALDI-TOF/MS. Two peaks (*m/z* 1022.33 and 1097.87) over 50% intensity were observed within the mass range from 1,000 Da to 10,000 Da, indicating small molecular weight peptides 10 amino acids long (Figure 3(b)).

Liquid chromatography tandem mass spectrometry (LC-MS/MS) was used to further identify the peptides. Output of the high-resolution MS/MS spectra, generated by the fragmentation of the precursor, with corresponding intensities was searched against the NCBI protein database of the taxonomy of Squamata (158856 entries, downloaded December 2016) using the Mascot search engine (version 2.2) (Table 2), revealing that the signals at* m/z* = 927.4531 [M+H+] and* m/z* = 769.38389,* m/z* = 851.42576,* m/z* = 615.30966, and* m/z* = 801.37372 [M+H+]+ corresponded to the single-protonated fragments of collagen alpha-1(I) chain from* Ophiophagus hannah*.

### 3.2. Effect of* Agkistrodon* Peptides Compound on Cell Viability and the mRNA Expression of TNF-*α*, IL-1*β*, and IL-6 in RAW264.7 Cells

To investigate the cytotoxicity of* Agkistrodon* peptides, the viability of RAW264.7 cells was evaluated by using the MTS assay (Figure 4(a)). Treatment with* Agkistrodon* peptides (0.1–100 *μ*g/mL) did not have a marked cytotoxic effect on the RAW264.7 cells. Furthermore, the anti-inflammatory effect was evaluated by stimulating the RAW264.7 cells with LPS. The stimulation of LPS promoted the expression of inflammatory cytokines at the mRNA level in RAW264.7 cells. However, treatment with* Agkistrodon* peptides suppressed the mRNA expression of TNF-*α*, IL-1*β*, and IL-6 in a dose-dependent manner (Figures 4(b)–4(d)).

### 3.3. Effect of* Agkistrodon* Peptides Compound on Paw Swelling and Ankle Changes in Groups with CIA

The effect of* Agkistrodon* peptides on CIA was evaluation with the hind paw swelling rate and arthritis index. The degree of swelling in the normal control group showed no significant increase. Compared with the normal control group, the degree of swelling in all other groups showed significant increases (data not shown). At the end of the experiment, compared with the model group the degree of swelling in the rats of treated groups showed significant reductions (*P* < 0.05 or *P* < 0.01). The degree of swelling in rats from the APE group showed a continuously significant reduction at days 9 and 21 of treatment (*P* < 0.01). The strong anti-inflammatory potency of the Chinese medicine* Tripterygium wilfordii* Hook (TWHF) has been verified [21–23], and the active component* Tripterygium glycoside* (GTW) [24–27] was used as the positive control in the present study.

Compared with those in either the GTW or APO group, those in the APE group had significantly reduced swelling. Additionally, there was no significant difference in paw volumes between the groups treated with GTW and APO (Figure 5(a)).

Visually apparent arthritis, with swollen joints in CIA rats, suggested that the CIA model was successfully established. At the end of the experiment, compared with the model group, all treated groups showed significant reductions (*P* < 0.05 or *P* < 0.01) in clinical arthritis indexes. Compared with the model group, a significant reduction was observed in the APE group. In addition, the arthritis index of the APE group was significantly different compared to the APO group (Figure 4(b)).

Plain radiographic analysis revealed normal bone structure with smooth and intact sharp and bone substance in the normal rats. However, signs of arthritis with soft-tissue swelling, uniform joint space narrowing, and bone damage were observed in the joints of rats in the model group. Compared with the model group, the soft-tissue swelling in treated groups was minimal, and the bone damage was not severe. In addition, markedly alleviated plain radiographic changes were observed in the joints of rats in the APE group (Figure 6(a)). Similar results showing the severe joint destruction were obtained with the three-dimensional images. The images showed smooth bone surface and intact joint architecture in normal rats and notable bone destruction in the CIA model rats while the bone destruction was much less robust in the APE group, suggesting that* Agkistrodon* peptides can protect bone integrity induced by CIA (Figure 6(b)).

### 3.4. Anti-Inflammatory Effect of* Agkistrodon* Peptides on Type II Collagen Antibodies Expression in Serum and TNF-*α*, IL-1*β*, and IL-6 Expression in Joints

The levels of anti-type II collagen IgG in treated groups was significantly reduced compared to the model group. The levels of anti-type II collagen IgG in the APE group were significantly lower than those in the GTW group. There was no significant difference between the APO and APE group (Figure 7).

Immunohistochemistry analysis revealed a higher expression of the inflammatory cytokines TNF-*α*, IL-1*β*, and IL-6 in the model group compared to the normal group. However, the expression level of these three cytokines were markedly reduced in the APE group compared to the model group or to the other treated groups, suggesting that* Agkistrodon* peptides could suppress the expression of the inflammatory cytokines TNF-*α*, IL-1*β*, and IL-6 under the arthritic conditions caused by CIA (Figures 8(a)–8(c)).

### 3.5. Improvement Effect of* Agkistrodon* Peptides on Pathological of Joints

The sections stained with H&E from the normal rats revealed that synovial cells on the surface of the ankle joints were well organized in the absence of inflammation. However, the images from rats in the model group revealed signs of severe arthritis with synovial hyperplasia, inflammatory cell infiltration, vascular pannus formation, and cartilage destruction. The extent of synovial hyperplasia and inflammatory cell infiltration in the joint of rats in the treated groups was comparatively reduced. In contrast, intact articular cartilage and slight inflammatory cell infiltration were observed in the joints of rats in the APE group (Figure 9(a)). A loss of safranin O and toluidine blue indicated severe cartilage destruction and bone erosion in rats from the model group. However, the damage was improved in the treated groups and the improvement was more prominent in the joints of rats from the APE group (Figures 9(b) and 9(c)). Collectively, these results demonstrated that* Agkistrodon* peptides can effectively attenuate inflammation, cartilage destruction, and bone erosion caused by CIA.

## 4. Discussion

**Figure 2:**
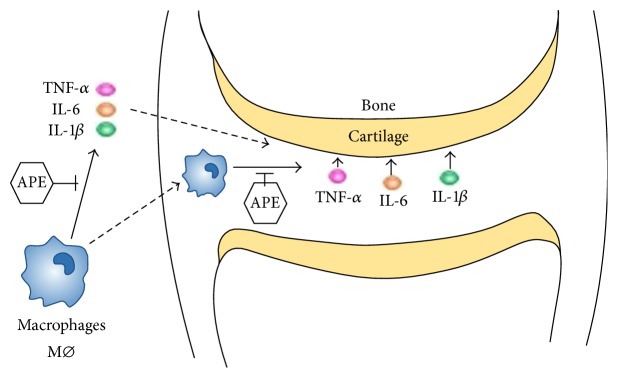
A schematic diagram of the effects and the principle molecular mechanisms of APE on Rheumatoid arthritis. APE reduces the recruitment of macrophages and thus downregulates the production of TNF-*α*, IL-6, and IL-1*β*, which are mainly released by macrophages.

**Figure 3:**
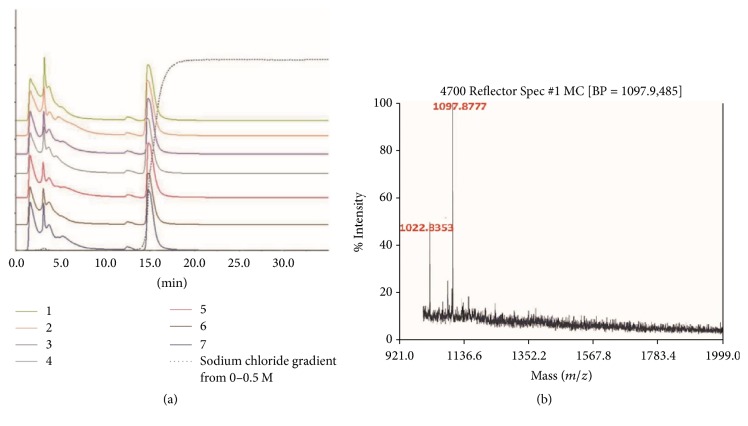
*Agkistrodon peptides isolation and identification*. (a) Seven repeats of fast separation of crude peptide mixture using ion-exchange chromatography. The dotted line refers to the sodium chloride gradient from 0 to 0.5 M. (b)* Agkistrodon* peptides identification by MALDI spectra.

**Figure 4:**
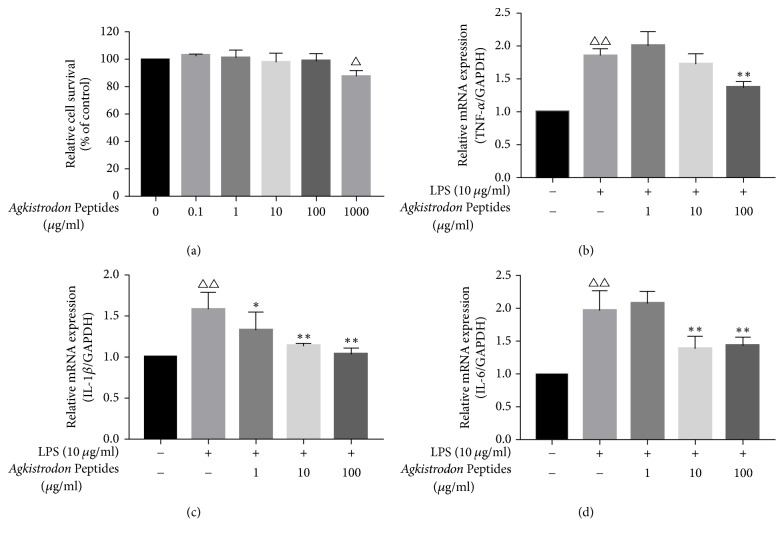
*Effect of Agkistrodon peptides on the inflammatory cytokines mRNA expression in RAW264.7 cells*. (a) The cytotoxic effect of* Agkistrodon* peptides on the viability of RAW264.7 cells. Cell viability was expressed as percentage of the control. Data were expressed as mean ± SD. ^△^*P* < 0.05 versus control. (b–d) The effect of* Agkistrodon* peptides on the mRNA expression levels of TNF-*α*, IL-1*β*, and IL-6 in LPS-stimulated RAW264.7 cells. Data were expressed as mean ± SD. ^△^*P* < 0.05; ^△△^*P* < 0.01 versus control. ^*∗*^*P* < 0.05; ^*∗∗*^*P* < 0.01 versus the LPS-treated cells.

**Figure 5:**
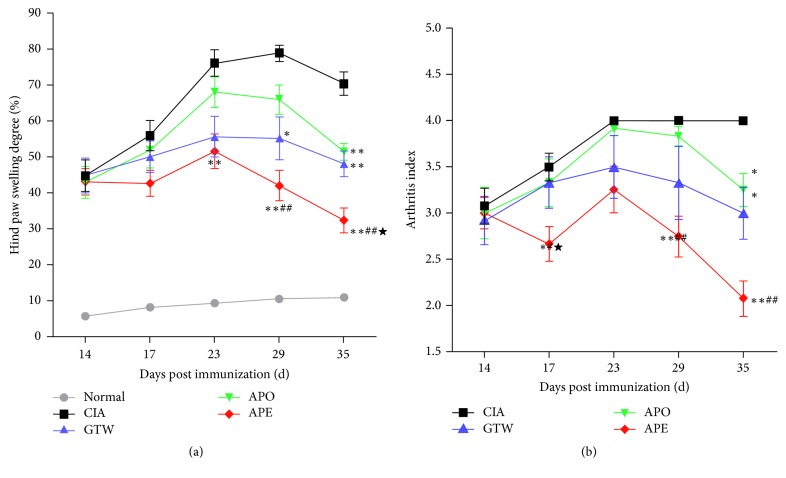
*Paw swelling changes in different groups of CIA*. (a) Paw swelling changes in different groups of CIA. Data were expressed as mean ± SEM (*n* = 12 hind paws). ^*∗*^*P* < 0.05; ^*∗∗*^*P* < 0.01 versus the model group. ^★^*P* < 0.05 versus the GTW group. ^#^*P* < 0.05; ^##^*P* < 0.01 versus the APE group. (b) Arthritis index changes in different groups of CIA. Data were expressed as mean ± SEM (*n* = 12 hind paws). ^*∗*^*P* < 0.05; ^*∗∗*^*P* < 0.01 versus the model group. ^★^*P* < 0.05 versus the GTW group. ^#^*P* < 0.05; ^##^*P* < 0.01 versus the APO group.

**Figure 6:**
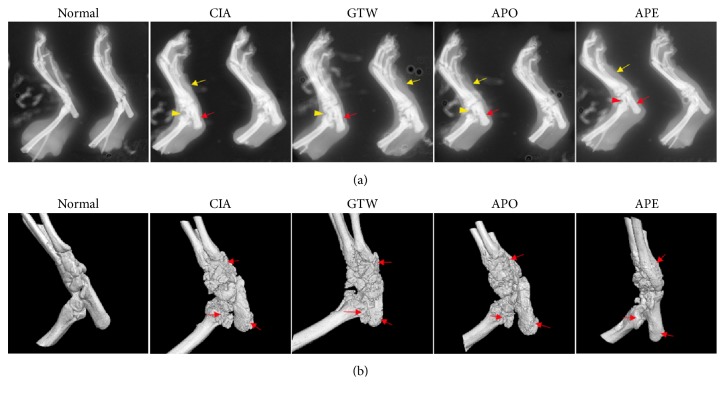
*Plain radiographs and three-dimensional images of rats' joints*. (a) The plain radiographs showed that soft-tissue swelling (yellow arrows), uniform joint space narrowing (triangles), and damaged bone substance (red arrows) were markedly alleviated in the joints of rats in the APE group. (b) The three-dimensional images showed that bone destruction (red arrows) was much less robust in the APE group compared with the model and other treated groups.

**Figure 7:**
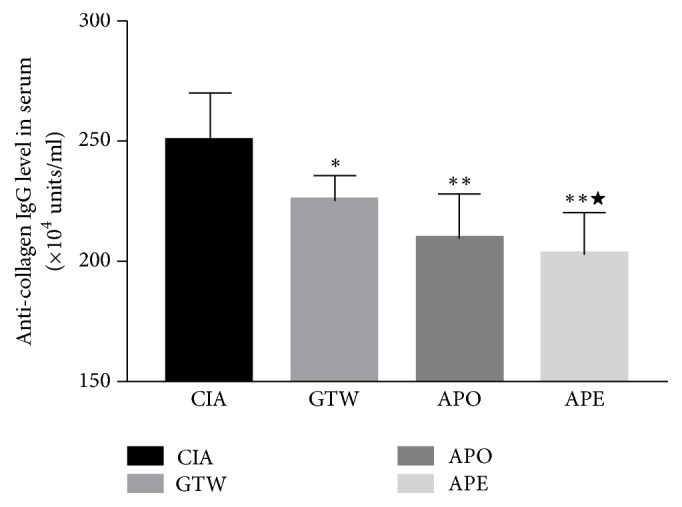
*Expression of collagen type II IgG in serum in different groups of CIA*. Data were expressed as mean ± SD. ^*∗*^*P* < 0.05; ^*∗∗*^*P* < 0.01 versus the model group. ^★^*P* < 0.05 versus the GTW group.

**Figure 8:**
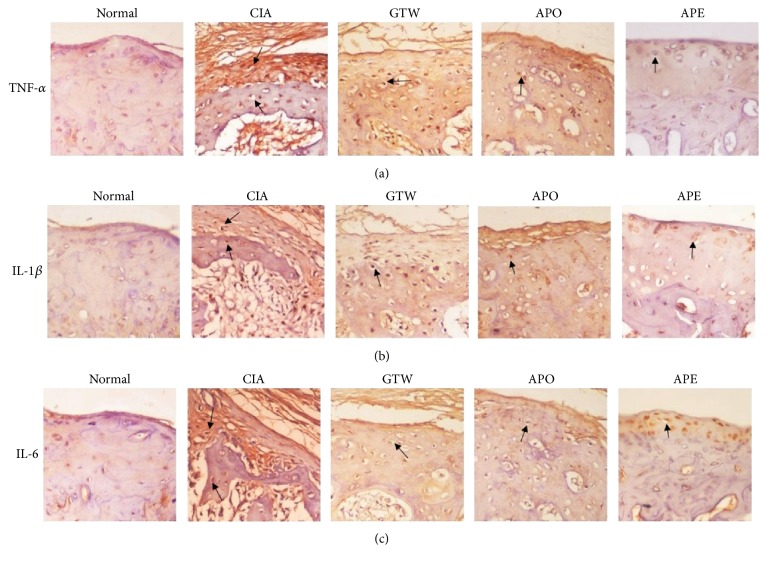
*Immunohistochemistry analysis in different groups of CIA*. (a) TNF-*α*, (b) IL-1*β*, and (c) IL-6 expression in joints (original magnification, ×200). The expressions of TNF-*α*, IL-1*β*, and IL-6 were decreased in joints of the treated groups compared with the model group. The arrows indicated locations of TNF-*α*, IL-1*β*, or IL-6.

**Figure 9:**
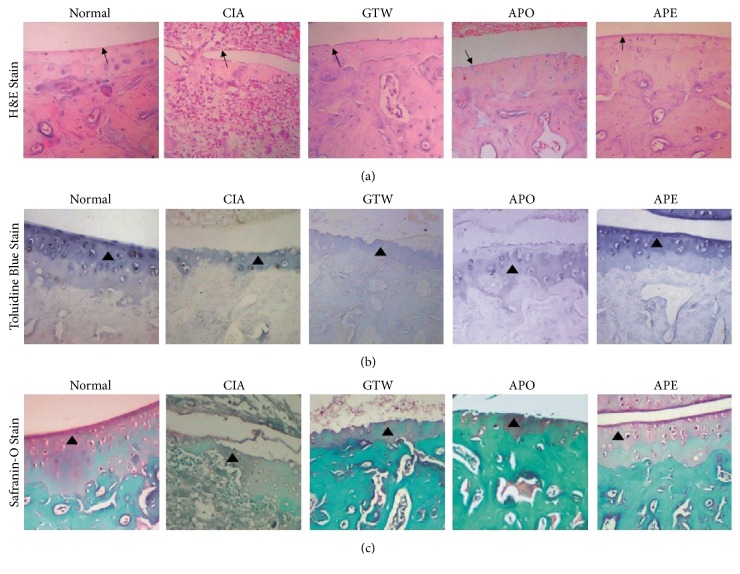
*Representative images of joint tissues stained with H&E, safranin O, and toluidine blue (original magnification, ×200)*. (a) Images stained with H&E revealed that synovial hyperplasia, inflammatory cell infiltration, the vascular pannus formation, and cartilage destruction were inhibited in the treated groups, especially the APE group when compared with the model group. The arrows indicated synovium. (b, c) Images stained with safranin O and toluidine blue showed that cartilage destruction and bone erosion were ameliorated in the treated groups, especially the APE group when compared with the model group. The triangles indicated articular cartilage.

Proinflammatory cytokines such as TNF-*α*, IL-1*β*, and IL-6 found in high concentrations in the synovial fluid and tissues of RA patients are crucial mediators of synovitis and subsequent erosive process of bone destruction. Macrophages release TNF-*α*, IL-6, and IL-1*β* to directly or indirectly act on inflammatory cells, leading to the inhibition of synthesis and the promotion of degradation of CII and chondrocyte apoptosis and may cause irreversible cartilage destruction and bone damage [2, 28, 29] (Figure 2). Alterations of proinflammatory cytokines have shown the potential for therapeutic intervention of RA [30, 31].

The* Agkistrodon* has long been used as a potent antiarthritic drug since ancient times [32]. A recent study carried out on CIA rats tested the optimal dosage of dried* Agkistrodon* for treatment of RA, yet functioning components within were not further discussed. In the present study, peptides were extracted from crude medicine of dried* Agkistrodon* and were further separated by ion-exchange chromatography (IEC), followed by MALDI-TOF/TOF mass spectrometry identification. Biological activity of the isolated peptides was tested. It was observed in RAW264.7 cells that LPS-stimulated TNF-*α*, IL-1*β*, IL-6, and NF-*κ*B mRNA expressions were inhibited in a dose-dependent manner. The peptides' effects were then evaluated by in vivo animal model of RA. The APE-treating group compared to the arthritis model group without had obviously lower clinical arthritis indexes and serum anti-collagen type II IgG. It was interesting to observe that the APE-treating group compared to the APO group had lower paw swelling and clinical arthritis indexes, which might be attributed to longer digestion time of the APO powder. While time was spared on digestion of the hard powder to release beneficial peptide/protein components, the small peptides compound may survive the degradation and exert an effect without the digestion time [33]. The peptides' antiarthritic effects were further supported by the histological HE staining results, demonstrating reduced synovial hyperplasia, inflammatory cell infiltration, and cartilage destruction, as well as the immunohistochemical assay that showed decreased expressions of TNF-*α*, IL-1*β*, and IL-6 in the rats' joints of the treating group.

Deviation of inflammatory cytokine production/secretion has been associated with protection mediated by different peptide epitopes of the autoantigens including heat shock protein 65, as well as collagen II [34, 35]. These tolerance-inducing peptides are believed to mediate suppressive effects by either directly neutralization of antigen-specific T cells or inducing cell populations that suppress the effector T cells [36, 37].

The mass spectrometer produced unique cleavage patterns of the peptides, which were then used to search for top matches within the database. It is interesting to note/notice that several fragments of the collagen from* Ophiophagus hannah* were recognized as matches, which strongly indicated the likelihood of existence of epitopes of the autoantigen. This might partially explain the inhibition of proinflammatory cytokines and the protection from bone destruction.

## 5. Conclusion

Previous study clarified the therapeutic benefits of the crude* Agkistrodon* powder, based on which we hypothesized peptides/proteins as vital components contained in the crude medicine. The hypothesis was confirmed in the present study by successful isolation of a small peptides compound, followed by demonstration of its better antiarthritic effect comparing the powder. The comparison implied potential of the small peptides as an equivalent therapy of the crude complex. Demonstration of the separation and beneficial effects of the compound though did not exclude the likelihood of potent peptides/proteins identified from the crude* Agkistrodon* in the near future by taking advantage of sophisticated chromatographic methods.

## Figures and Tables

**Table 1:** Primers for the target genes used in RT-PCR.

Target gene	Primer	Length of target fragment (bp)
TNF-*α*	5′-CCACATCTCCCTCCAGAAAAGA -3′ (F)	768
	5′-GCTGGGTAGAGAATGGATGAAC -3′ (R)	
IL-1*β*	5′-GCTTCAGGCAGGCAGTAT -3′ (F)	472
	5′-ACAAACCGCTTTTCCATCT -3′ (R)	
IL-6	5′-GAAATCGTGGAAATGAG -3′ (F)	455
	5′-TAGGTTTGCCGAGTAGA -3′ (R)	
GAPDH	5′-TGCACCACCAACTGCTTAG -3′ (F)	177
	5′-GGATGCAGGGATGATGTTC -3′ (R)	

**Table 2:** Separated peptide fractions of *Agkistrodon* identified by Mascot.

Number	Sequence	MW (MH+)	Diff (MH+)	PI	Score	*P* value
1	R.GPPGSSGSPGK.D	927.45305	−0.00007	8.75	92.7	6.08*E* − 08
2	K.GDAGPAGPK.G	769.38389	−0.00022	5.84	84.55	4.16*E* − 07
3	R.GFSGLDGAK.G	851.42576	0.00022	5.84	75.76	4.13*E* − 06
4	R.DGAAGPK.G	615.30966	−0.00008	5.84	66.96	1.37*E* − 05
5	K.GEPGDAGAK.G	801.37372	0.00048	4.37	60.84	5.36*E* − 05
